# Mortality and Respiratory-Related Hospitalizations in Idiopathic Pulmonary Fibrosis Not Treated With Antifibrotics

**DOI:** 10.3389/fmed.2021.802989

**Published:** 2021-12-24

**Authors:** Vincent Cottin, Paolo Spagnolo, Philippe Bonniaud, Maëva Nolin, Faustine Dalon, Klaus-Uwe Kirchgässler, Tripthi V. Kamath, Eric Van Ganse, Manon Belhassen

**Affiliations:** ^1^National French Reference Coordinating Center for Rare Pulmonary Diseases, Louis Pradel Hospital and Hospices Civils de Lyon, Université de Lyon, Université Claude Bernard Lyon 1, INRAE, IVPC, ERN-LUNG, Lyon, France; ^2^Respiratory Disease Unit, Department of Cardiac, Thoracic, Vascular Sciences and Public Health, University of Padova, Padova, Italy; ^3^Service de Pneumologie et Soins Intensifs Respiratoires, Centre de Référence Constitutif des Maladies Pulmonaires Rares de l'Adulte, Centre Hospitalo-Universitaire de Bourgogne and Faculté de Médecine et Pharmacie, Université de Bourgogne-Franche Comté, Dijon, France; ^4^INSERM U123-1, Dijon, France; ^5^PharmacoEpidemiology Lyon (PELyon), Lyon, France; ^6^F. Hoffmann-La Roche, Ltd., Basel, Switzerland; ^7^Genentech, Inc., South San Francisco, CA, United States; ^8^Respiratory Medicine, Croix Rousse University Hospital and Research on Healthcare Performance (RESHAPE), INSERM U1290, Université Claude Bernard Lyon 1, Lyon, France

**Keywords:** idiopathic pulmonary fibrosis, antifibrotics, mortality, acute hospitalizations, claims data

## Abstract

**Background:** Real-world data regarding outcomes of idiopathic pulmonary fibrosis (IPF) are scarce, outside of registries. The claims data from the French National Health System (SNDS) were used to describe outcomes in patients diagnosed with IPF in 2015–2016 but who did not receive antifibrotic therapies.

**Method:** Patients aged <50 years were excluded, as were patients with pulmonary fibrosis other than IPF, patients who had previously received a lung transplant, and those who had received antifibrotic therapies at any time between 2010 and 2016. Patients were followed-up until their last health record, lung transplantation, initiation of antifibrotic therapies, death, or the end of the study period (31 December 2017), whichever occurred first.

**Results:** A total of 5,360 patients (43.2%) not treated with antifibrotic therapies were included. The mean age was 75.5 years, and 57.9% were males. In the year before inclusion, 47.3% of patients had a Charlson score ≥5. During follow-up, 41.2% of patients died. The unadjusted incidence rate was 29.9 per 100 person-years (95%CI = [28.7–31.2]), and the cumulative incidence of death at 3 years was 50.2% (95% CI = [48.3–52.1%]). In the study population, 35.3% of patients experienced an acute respiratory-related hospitalization. The unadjusted incidence rate was 32.1 per 100 person-years (95%CI = [30.6–33.5]) and the cumulative incidence of the event at 3 years was 41.5% (95% CI = [39.7–43.2%]).

**Interpretation:** This observational study showed that, if untreated with antifibrotics, IPF is associated with a 50% all-cause mortality at 3 years. These figures can serve as a historical control of the natural course of the disease.

## Introduction

Idiopathic pulmonary fibrosis (IPF) is a chronic, relentlessly progressive and ultimately fatal lung disease of unknown etiology (1) leading to death within 2–5 years of diagnosis if untreated, often with interspersed episodes of acute exacerbation (2). Pirfenidone and nintedanib, currently the only approved antifibrotic therapies for IPF (3), slow lung function decline and reduce the risk of respiratory-related hospitalizations, which are associated with high morbidity and mortality (4, 5). Nonetheless, many individuals diagnosed with IPF do not receive antifibrotic therapies (6). Nowadays, real world survival data from large unselected and untreated IPF patient populations outside of registries are scarce. Here, using claims data from the French National Health System (NHS), we studied the characteristics, all-cause mortality, and rates of acute respiratory-related hospitalizations of patients diagnosed with IPF but who did not receive antifibrotics.

## Methods

This retrospective, population-based cohort study used digital data from the Système National des Données de Santé (SNDS), which covers 98.8% of the population living in France. This unique real-world dataset of French healthcare utilization is one of the largest data repositories worldwide. It contains comprehensive, anonymous, individual information on sociodemographic characteristics, date of death, out-of-hospital reimbursed healthcare expenditures (from both public and private healthcare), and hospital discharge summaries with International Classification of Diseases (ICD)-10 codes (7). In addition, the SNDS contains direct information on medical diagnoses for patients who have full coverage by the NHS for all medical expenses (“chronic disease status”), including most patients diagnosed with IPF in France.

The study population consisted of patients diagnosed with IPF identified in the SNDS between 1 January 2015 and 31 December 2016. Patients were included if they were hospitalized for IPF as the main or related diagnosis (J841 ICD-10 code), or had a “chronic disease” status for IPF. To ensure analytical data exhaustivity, only patients continuously covered by the French NHS during the study period (between 1 January 2010 and 31 December 2017) were included.

Patients younger than 50 were excluded, as well as patients with pulmonary fibrosis other than IPF (see Supplementary Table 1), patients who had previously received a lung transplant, and those who had received antifibrotic therapies at any time between 2010 and 2016 (8). Patients were followed-up until their last health record, lung transplantation, initiation of antifibrotics, death, or the end of the study period (31 December 2017), whichever occurred first. Pirfenidone has been available in France since October 2012 and nintedanib since April 2015. As data from SNDS are made available with some delay, 2017 was the last year available at the time of the analyses and follow-up was censored as of 31 December 2017.

The outcomes of interest were all-cause mortality and acute respiratory-related hospitalization, i.e., related to acute respiratory events, triggered or idiopathic, as identified using the main diagnoses of hospitalizations [see Supplementary Table 2; (8)]. Time to first occurrence of the events was estimated using cumulative incidence functions with confidence intervals. For each patient, differences in follow-up were accounted for through non-informative censoring at the end of follow-up. As mortality was >10%, the competing risk of mortality was accounted for through informative censoring at death.

All statistical analyses were performed using SAS (SAS Institute, North Carolina, US), version 9.4.

This study was approved by the French Institute for Health Data (approval no. 57932 from 12 July 2018). It was conducted with anonymised data, as requested by the National Informatics and Liberty Commission (CNIL), approval no. 918255, from 8 August 2018.

## Results

**Figure 1:**
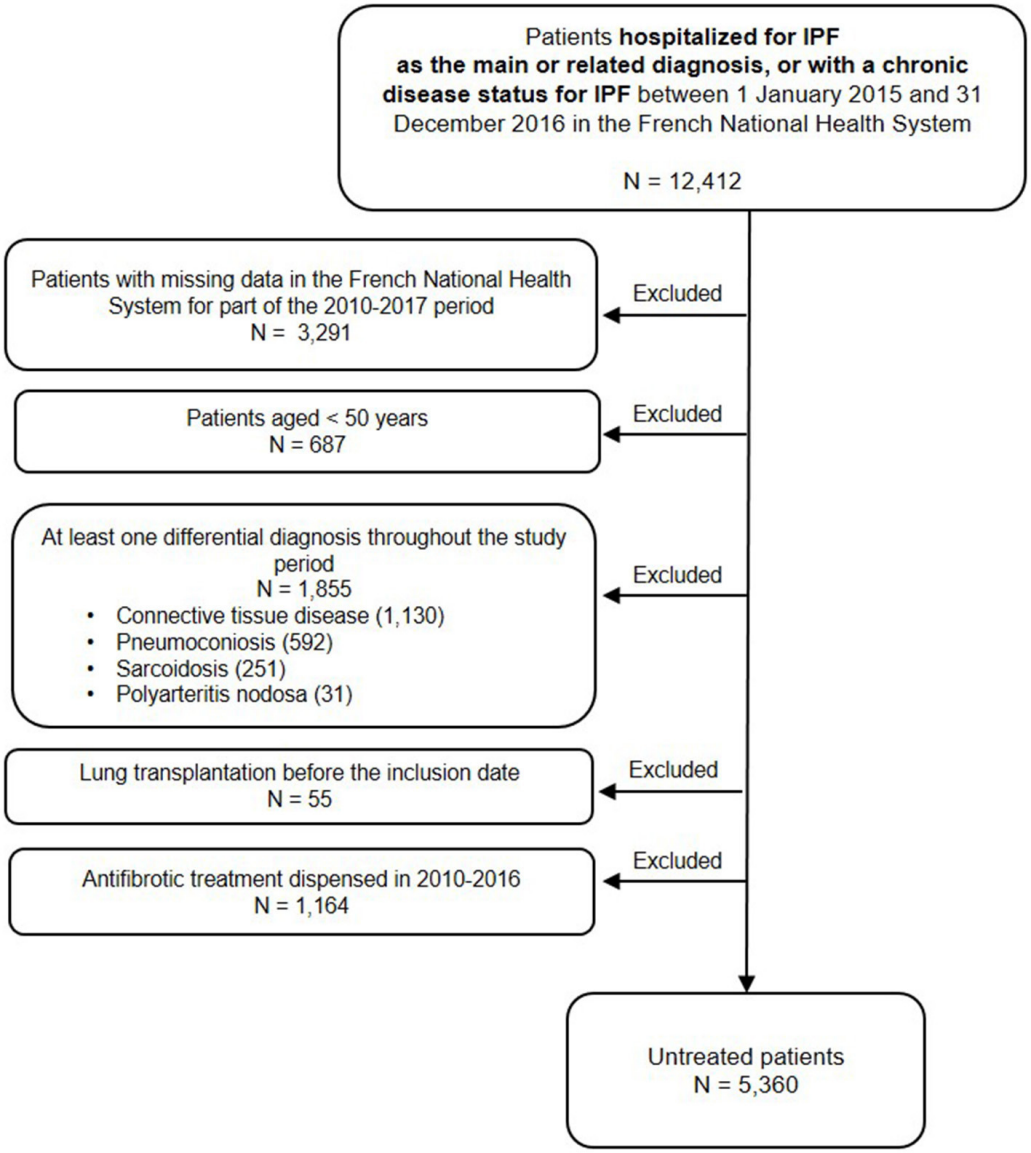
Study flowchart.

Among the 12,412 patients hospitalized for IPF or with a chronic disease status for IPF between 2015 and 2016, 7,052 patients were excluded: 3,291 patients (26.5%) who had no continuous follow-up during the 2010–2017 period (i.e., patients with no exhaustive data in the database), 687 patients (5.5%) because they were <50 years old, 1,855 patients (14.9%) who presented at least one differential diagnosis, 55 patients (0.4%) who had a lung transplantation before the inclusion date, and 1,164 patients (9.4%) who received at least one dispensing of antifibrotic therapies between 2010 and 2016. A total of 5,360 patients (43.2%) not treated with antifibrotic therapies were included (Figure 1).

The mean age was 75.5 years, and 57.9% were males. In the year before inclusion, 47.3% of patients had a Charlson score ≥5, 74% of patients visited an office-based lung specialist or a hospital practitioner at least once, 81.7% of patients experienced at least one acute respiratory-related hospitalizations, and 19.4% of patients used supplementary oxygen (Table 1).

**Table 1:** Baseline characteristics of IPF patients.

	**Untreated patients (*N* = 5,360)**
**Gender**, ***n*** **(%)**	
Male	3,101 (57.9%)
Female	2,259 (42.1%)
**Age at index date (in years)**	
Mean (SD)	75.5 (10.6)
Median (IQR)	77.0 (68.0–84.0)
Min – Max	50.0–102.0
**Number of acute respiratory-related hospitalization**	
0	983 (18.3%)
1	3,481 (64.9%)
2 or more	896 (16.7%)
**Number of visits to office-based lung specialists or hospital**	
**practitioners (all specialties combined)**	
No visit	1,395 (26.0%)
One to two visits	1,875 (35.0%)
Three to four visits	989 (18.5%)
Five or more visits	1,101 (20.5%)
**Comorbidities in the year before inclusion**	
Depression and anxiety	2,554 (47.6%)
Chronic obstructive pulmonary disease	1,575 (29.4%)
Hypertensive diseases	1,504 (28.1%)
Other forms of heart disease	1,259 (23.5%)
Diabetes mellitus	1,181 (22.0%)
Ischemic heart diseases	1,050 (19.6%)
Heart failure	797 (14.9%)
Disorders of lipoprotein metabolism and other lipidemias	412 (7.7%)
Pulmonary hypertension	260 (4.9%)
Sleep apnea	250 (4.7%)
Emphysema	221 (4.1%)
Malignant neoplasm of bronchus and lungs	182 (3.4%)
Pulmonary embolism	127 (2.4%)
Other diseases of pulmonary vessels	1 (0.02%)
**Charlson score**	
1–2	743 (13.9%)
3–4	2,082 (38.8%)
≥5	2,535 (47.3%)
**Presence of oxygen therapy**	
No	4,319 (80.6%)
Yes	1,041 (19.4%)

**Figure 2:**
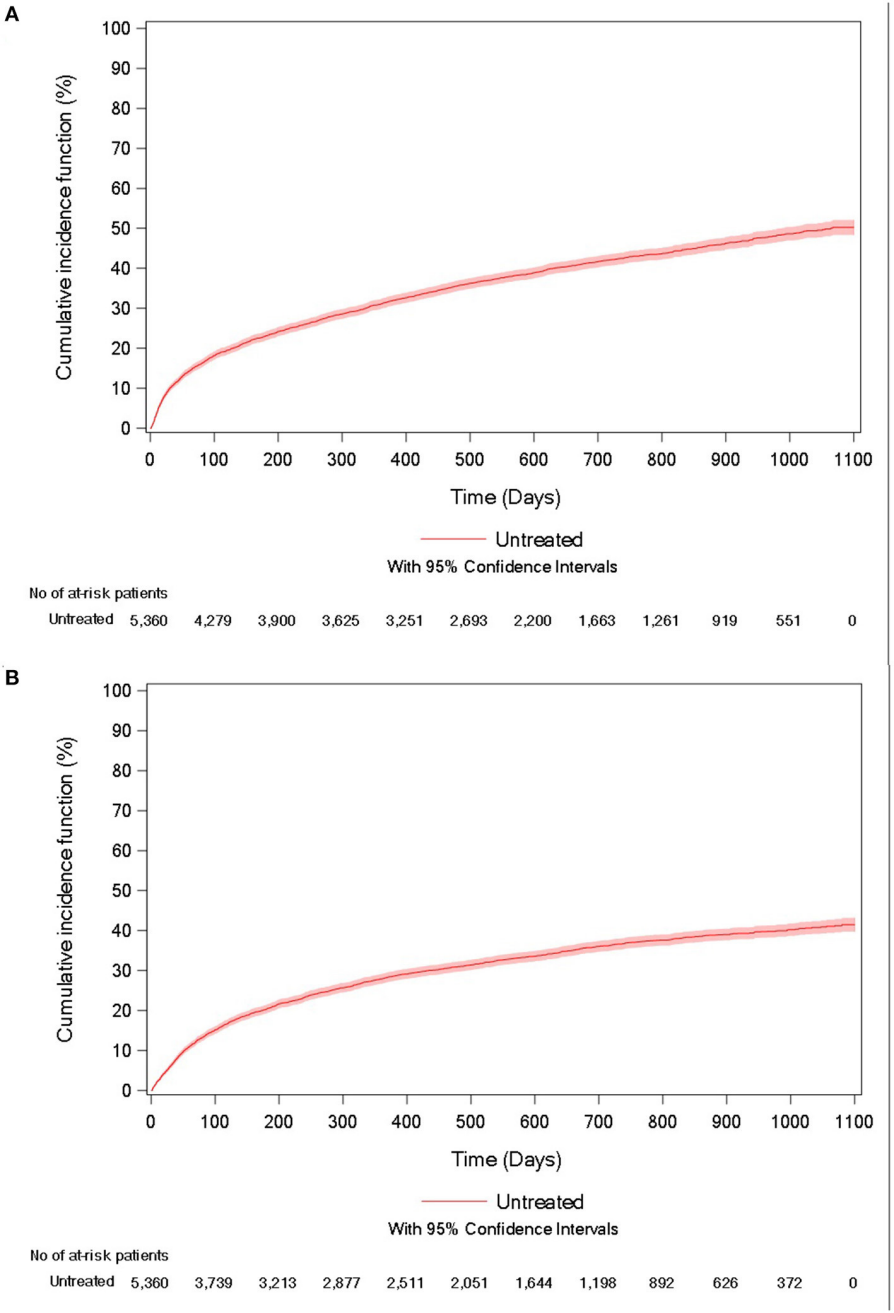
Cumulative incidence curve of **(A)** all-cause mortality and **(B)** acute respiratory-related hospitalization for untreated IPF patients.

During follow-up, 41.2% of patients died, with a mean of 236.2 days (247.4) between the inclusion date and death. The unadjusted incidence rate was 29.9 per 100 person-years (95%CI = [28.7–31.2]), and the cumulative incidence of death at 3 years was 50.2% (95% CI = [48.3–52.1%]; Figure 2A).

In the study population, 35.3% of patients experienced an acute respiratory-related hospitalization, with a mean of 223.2 days (228.3) between the inclusion date and the event. The unadjusted incidence rate was 32.1 per 100 person-years (95%CI = [30.6–33.5]) and the cumulative incidence of the event at 3 years was 41.5% (95% CI = [39.7–43.2%]; Figure 2B).

## Discussion

In summary, IPF patients untreated with antifibrotic therapies had poor outcomes, with a 50% all-cause mortality and a 42% cumulative incidence rate of acute respiratory-related hospitalization at 3 years. Using the same dataset, we previously found cumulative incidence rates at 3 years of 26 and 31% for all-cause mortality, and of 48 and 44% for acute respiratory-related hospitalization, in patients newly treated with pirfenidone and nintedanib, respectively (8).

These findings confirm, in an unselected cohort of 5,360 patients, the progressive and ultimately fatal nature of the disease, in agreement with the literature (9, 10). In a study by Dempsey et al. (11), all-cause mortality cumulative risk was 16.5% (14.2–19.1) at 52 weeks, but their study included younger and less severe patients than ours. In a study by Nathan et al. (12) and the INPULSIS trial (5), 6.7 and 7.8% of the patients died in the placebo group, respectively, confirming lower mortality rates in Phase 3 trials compared to clinical practice, possibly as a result of the selection of less severe IPF patients in trials.

We computed all-cause mortality in IPF patients untreated with antifibrotic therapies, a major endpoint for both patients and clinicians (13). Indeed, the French health care utilization dataset contains exhaustive individual data on drug use, hospital admissions, and death, therefore producing robust figures on either use -or non-use- of therapy and outcomes. The specificities of the French health care system -a single payer system covering the entire population-, and the completeness and duration of individual records of health care utilization make it a valid tool for investigating IPF and specific therapy in the overall population.

Similar to other investigations of this kind, this study is limited by its observational design and its data source. The ICD-10 code used to confirm the presence of pulmonary fibrosis (J841) is not specific of IPF, and ICD codes and age were mostly used to exclude other forms of fibrotic interstitial lung diseases among patients initiating an antifibrotic therapies. Patients younger than 50 years were not included, therefore patients with IPF of genetic origin may be underrepresented. Although imperfect, this approach has previously been used in other studies on claims data and in a study using the French healthcare dataset that further validated the definition algorithm for acute respiratory-related hospitalizations (11, 14). Data on lung function, a major determinant of IPF prognosis, were not available, as is mostly the case for claims data. There was a high rate of patients not receiving an antifibrotic therapy in this study, comparable to other studies (11), with multiple possible causes, including the study period and relative novelty of antifibrotic therapies at the time; patients' and physicians' preferences; and the small number of ILD centers in France at the time (15). Reasons for not initiating antifibrotic therapies were however not available. The proportion of patients diagnosed with IPF who now receive an antifibrotic therapies may have increased. The rate of patients receiving supplemental oxygen therapy was relatively low however consistent with another study (16). In conclusion, this observational study that included over 5,000 IPF patients showed that, if untreated with antifibrotic therapies, the disease is associated with a 50% all-cause mortality at 3 years. These figures can serve as a historical control of the natural course of the disease.

## Data Availability Statement

The data analyzed in this study is subject to the following licenses/restrictions: Due to NHS and SNDS rules, no data sharing is possible as access to data is restricted to habilitated and qualified researchers.

## Author Contributions

VC, MB, and EVG were responsible for producing the initial draft of the paper. MN carried out the primary statistical analysis and including figures and tables. PS, PB, FD, K-UK, and TK provided written comments and feedback during manuscript development and were directly involved in the execution of the study. All authors contributed to the article and approved the submitted version.

## Funding

This work was supported by F. Hoffmann-La Roche, Ltd. Medical writing support was provided by PELyon, funded by F. Hoffmann-La Roche, Ltd.

## Conflict of Interest

FD, MN, and MB are full-time employees of PELyon. EVG is the scientific advisor of PELyon. VC reports personal fees and non-financial support from Actelion, Bayer/MSD, and Roche; grants, personal fees, and non-financial support from Boehringer Ingelheim; personal fees from Novartis, Sanofi, Promedior, Celgene, Galapagos, and Galecto, outside the submitted work. PS reports institutional grants, consulting fees, and non-financial support from PPM Services; institutional grants, personal fees, and non-financial support from Roche and Boehringer Ingelheim; personal fees from Chiesi, Galapagos, Lupin, Pieris, and REDX Pharma, outside the submitted work. K-UK is an employee and shareholder of F. Hoffmann-La Roche, Ltd. PB reports personal fees and non-financial support from Roche, Boehringer Ingelheim, Novartis, Sanofi, Chiesi, AstraZeneca, Stallergenes, and GSK, outside the submitted work. TK is a full-time employee at Janssen Pharmaceuticals. The reviewer NB declared a shared affiliation with one of the author PS, to the handling editor at time of review.

## Publisher's Note

All claims expressed in this article are solely those of the authors and do not necessarily represent those of their affiliated organizations, or those of the publisher, the editors and the reviewers. Any product that may be evaluated in this article, or claim that may be made by its manufacturer, is not guaranteed or endorsed by the publisher.

## References

[1] Richeldi L Collard HR Jones MG. Idiopathic pulmonary fibrosis. Lancet. (2017) 389:1941–52. 10.1016/S0140-6736(17)30866-8 28365056

[2] Ley B Collard HR King TE Jr. Clinical course and prediction of survival in idiopathic pulmonary fibrosis. Am J Respir Crit Care Med. (2011) 183:431–40. 10.1164/rccm.201006-0894CI 20935110

[3] Cottin V Crestani B Cadranel J Cordier JF Marchand-Adam S Prevot G. French practical guidelines for the diagnosis and management of idiopathic pulmonary fibrosis - 2017 update. Full-length version. Rev Mal Respir. (2017) 34:900–68. 10.1016/j.rmr.2017.07.022 28939155

[4] Noble PW Albera C Bradford WZ Costabel U Glassberg MK Kardatzke D. Pirfenidone in patients with idiopathic pulmonary fibrosis (CAPACITY): two randomised trials. Lancet. (2011) 377:1760–9. 10.1016/S0140-6736(11)60405-4 21571362

[5] Richeldi L du Bois RM Raghu G Azuma A Brown KK Costabel U. Efficacy and safety of nintedanib in idiopathic pulmonary fibrosis. N Engl J Med. (2014) 370:2071–82. 10.1056/NEJMoa1402584 24836310

[6] Maher TM Molina-Molina M Russell AM Bonella F Jouneau S Ripamonti E. Unmet needs in the treatment of idiopathic pulmonary fibrosis-insights from patient chart review in five European countries. BMC Pulm Med. (2017) 17:124. 10.1186/s12890-017-0468-5 28915874 PMC5602932

[7] Tuppin P Rudant J Constantinou P Gastaldi-Menager C Rachas A de Roquefeuil L. Value of a national administrative database to guide public decisions: from the systeme national d'information interregimes de l'Assurance Maladie (SNIIRAM) to the systeme national des donnees de sante (SNDS) in France. Rev Epidemiol Sante Publique. (2017) 65(Suppl. 4):S149–67. 10.1016/j.respe.2017.05.004 28756037

[8] Belhassen M Dalon F Nolin M Van Ganse E. Comparative outcomes in patients receiving pirfenidone or nintedanib for idiopathic pulmonary fibrosis. Respir Res. (2021) 22:135. 10.1186/s12931-021-01714-y 33947414 PMC8094468

[9] Maher TM Strek ME. Antifibrotic therapy for idiopathic pulmonary fibrosis: time to treat. Respir Res. (2019) 20:205. 10.1186/s12931-019-1161-4 31492155 PMC6731623

[10] Ryerson CJ Kolb M. The increasing mortality of idiopathic pulmonary fibrosis: fact or fallacy? Eur Respir J. (2018) 51:1702420. 10.1183/13993003.02420-2017 29348187

[11] Dempsey TM Sangaralingham LR Yao X Sanghavi D Shah ND Limper AH. Clinical effectiveness of antifibrotic medications for idiopathic pulmonary fibrosis. Am J Respir Crit Care Med. (2019) 200:168–74. 10.1164/rccm.201902-0456OC 31150266

[12] Nathan SD Albera C Bradford WZ Costabel U Glaspole I Glassberg MK. Effect of pirfenidone on mortality: pooled analyses and meta-analyses of clinical trials in idiopathic pulmonary fibrosis. Lancet Respir Med. (2017) 5:33–41. 10.1016/S2213-2600(16)30326-5 27876247

[13] Raghu G Collard HR Anstrom KJ Flaherty KR Fleming TR King TE Jr. Idiopathic pulmonary fibrosis: clinically meaningful primary endpoints in phase 3 clinical trials. Am J Respir Crit Care Med. (2012) 185:1044–8. 10.1164/rccm.201201-0006PP 22505745 PMC5448580

[14] Cottin V Schmidt A Catella L Porte F Fernandez-Montoya C Le Lay K. Burden of idiopathic pulmonary fibrosis progression: a 5-year longitudinal follow-up study. PLoS ONE. (2017) 12:e0166462. 10.1371/journal.pone.0166462 28099456 PMC5242514

[15] Cottin V Cadranel J Crestani B Dalphin JC Delaval P Israel-Biet D. Management of idiopathic pulmonary fibrosis in France: a survey of 1244 pulmonologists. Respir Med. (2014) 108:195–202. 10.1016/j.rmed.2013.11.017 24361163

[16] Tran T Šterclová M Mogulkoc N Lewandowska K Müller V Hájková M. The European MultiPartner IPF registry (EMPIRE): validating long-term prognostic factors in idiopathic pulmonary fibrosis. Respir Res. (2020) 21:11. 10.1186/s12931-019-1271-z 31915023 PMC6951015

