# Supplementary material for: Mortality and Respiratory-Related Hospitalizations in Idiopathic Pulmonary Fibrosis Not Treated With Antifibrotics
Source: Front Med (Lausanne). 2021 Dec 24;8:802989. doi: 10.3389/fmed.2021.802989 (PMC8739228; doi:10.3389/fmed.2021.802989)
Supplement: Supplementary file 1 [file Table_1.DOCX]

**Additional tables**

**Additional table 1** ICD-10 codes for exclusion criteria

| **Diagnosis** | **Label** | **Hospitalisation** | **CD status** |
| --- | --- | --- | --- |
| Connective tissue diseases | Rheumatoid lung disease | J990 | – |
|  | Rheumatoid lung disease | M051 | – |
|  | Other necrotising vasculopathies | M31 | M31 |
|  | Systemic lupus erythematosus | M32 | M32 |
|  | Dermatopolymyositis | M33 | M33 |
|  | Systemic sclerosis | M34 | M34 |
|  | Other systemic involvement of connective tissue | M35 | M35 |
|  | Other localised connective tissue disorders | L94 | L94 |
| Pneumoconiosis | Coalworker pneumoconiosis | J60 | J60 |
|  | Pneumoconiosis due to asbestos and other mineral fibres | J61 | J61 |
|  | Pneumoconiosis due to dust containing silica | J62 | J62 |
|  | Pneumoconiosis due to other inorganic dust | J63 | J63 |
|  | Unspecified pneumoconiosis | J64 | J64 |
|  | Pneumoconiosis associated with tuberculosis | J65 | J65 |
|  | Airway disease due to specific organic dust | J66 | J66 |
|  | Hypersensitivity pneumonitis due to organic dust | J67 | J67 |
|  | Pleural plaque with presence of asbestos | J920 | – |
| Sarcoidosis | Sarcoidosis | D86 | D86 |
| Polyarteritis nodosa | Polyarteritis nodosa and related conditions | M30 | M30 |

CD: chronic disease; ICD-10: 10th revision of the International Statistical Classification of Diseases and Related Health Problems.

**Additional table 2** ICD-10 codes for acute respiratory-related hospitalisations (main diagnosis)

| **Diagnosis** | **Label** | **Hospitalisation** |
| --- | --- | --- |
| Legionnaires’ disease | Legionnaires’ disease | A481 |
| HIV disease resulting in infectious and parasitic diseases | HIV disease resulting in *Pneumocystis carinii* pneumonia | B206 |
| Cytomegaloviral pneumonitis | Cytomegaloviral pneumonitis | B250 |
| Pneumocystosis | Pneumocystosis | B59 |
| Influenza | Influenza due to identified zoonotic or pandemic influenza virus | J09 |
|  | Influenza due to identified seasonal influenza virus | J10 |
|  | Influenza, virus not identified | J11 |
| Pneumonia | Viral pneumonia, not classified elsewhere | J12 |
|  | Pneumonia due to *Streptococcus pneumoniae* | J13 |
|  | Pneumonia due to *Haemophilus influenzae* | J14 |
|  | Bacterial pneumonia not classified elsewhere | J15 |
|  | Pneumonia due to other infectious organisms not classified elsewhere | J16 |
|  | Pneumonia in diseases classified elsewhere | J17 |
|  | Pneumonia, organism unspecified | J18 |
| Bronchitis (not specified as acute or chronic) | Bronchitis not specified as acute or chronic | J40 |
| Bronchitis | Acute bronchitis | J20 |
| Acute bronchiolitis | Acute bronchiolitis | J21 |
| Unspecified acute lower respiratory infection | Unspecified acute lower respiratory infection | J22 |
| Other interstitial pulmonary diseases with fibrosis | Other interstitial pulmonary diseases with fibrosis | J841 |
| Pneumothorax | Pneumothorax | J93 |
| Acute respiratory failure | Acute respiratory failure | J960 |
| Chronic respiratory failure | Chronic respiratory failure | J961 |
| Unspecified respiratory failure | Respiratory failure, unspecified | J969 |

HIV: human immunodeficiency virus; ICD-10: 10th revision of the International Statistical Classification of Diseases and Related Health Problems.
